# Expanding the use of real‐time electromagnetic tracking in radiation oncology

**DOI:** 10.1120/jacmp.v12i4.3590

**Published:** 2011-11-15

**Authors:** Amish P. Shah, Patrick A. Kupelian, Twyla R. Willoughby, Sanford L. Meeks

**Affiliations:** ^1^ Department of Radiation Oncology MD Anderson Cancer Center Orlando Orlando Florida 32806

**Keywords:** electromagnetic tracking, Calypso, localization

## Abstract

In the past 10 years, techniques to improve radiotherapy delivery, such as intensity‐modulated radiation therapy (IMRT), image‐guided radiation therapy (IGRT) for both inter‐ and intrafraction tumor localization, and hypofractionated delivery techniques such as stereotactic body radiation therapy (SBRT), have evolved tremendously. This review article focuses on only one part of that evolution, electromagnetic tracking in radiation therapy. Electromagnetic tracking is still a growing technology in radiation oncology and, as such, the clinical applications are limited, the expense is high, and the reimbursement is insufficient to cover these costs. At the same time, current experience with electromagnetic tracking applied to various clinical tumor sites indicates that the potential benefits of electromagnetic tracking could be significant for patients receiving radiation therapy. Daily use of these tracking systems is minimally invasive and delivers no additional ionizing radiation to the patient, and these systems can provide explicit tumor motion data. Although there are a number of technical and fiscal issues that need to be addressed, electromagnetic tracking systems are expected to play a continued role in improving the precision of radiation delivery.

PACS number: 87.63.‐d

## I. INTRODUCTION

Electromagnetic tracking has been in use for a number of medical applications including image‐guided interventional therapy and surgery, endoscopic navigation and more recently, localization and tracking systems for prostate radiotherapy. For the past 15 years, development of electromagnetic tracking systems has grown from an early need in surgical navigation to a more recent need for precision radiotherapy. Early image‐guided surgery and radiation therapy systems relied on optical tracking. Optical tracking systems have limitations, however, that have led to increased interest in electromagnetic tracking systems for medical use. Optical tracking systems require a line‐of‐sight between light‐emitting diodes and tracking system cameras, whereas electromagnetic systems do not. Electromagnetic systems can be used in computer‐aided medical procedures by defining position and orientation for guidewires in interventional radiology or catheter placement for bronchoscopic procedures.

Several electromagnetic tracking systems have been developed for image‐guided surgery using wired transponders such as the Aurora (Northern Digital Inc., Waterloo, Ontario) and microBIRD systems (Ascension Technology Corporation, Burlington, VT). Another similar system has been developed by SuperDimension Inc. (Minneapolis, Minnesota) in order to guide endoscopic tools and catheters down the pulmonary tract so as to provide clinicians the ability to navigate to lesions and biopsy for later diagnosis. This electromagnetic technology has also been used in radiation oncology to guide the implantation of radiosurgical markers or fiducials in and around peripheral lung lesions.^(^ ^1^ ^)^

At the same time, the need for accurate daily targeting during a course of external beam radiotherapy in the treatment of localized malignancies has also led to advancements in electromagnetic tracking systems. These electromagnetic tracking systems provide the clinician with previously unavailable real‐time motion information for targets that may have substantial motion. Currently, the most prevalent use of electromagnetic tracking technology in radiation therapy is with the localization and tracking system offered through Calypso Medical Technologies (Calypso Medical Technologies, Inc. Seattle, WA). This system is currently approved by the Food and Drug Administration (FDA) for use in prostate and post‐prostatectomy prostate bed radiation therapy. Other similar electromagnetic tracking systems for linear accelerator radiotherapy have been developed by Micropos Medical (Sweden) and Northern Digital Inc. (Waterloo, Canada), but these are investigational or prototype devices and are not cleared for sale in the United States for use as tumor tracking devices in radiation oncology. We will discuss their theory of operation, as well as the forecast for electromagnetic tracking including innovations, clinical indications, and challenges within radiation oncology.

## II. CURRENT STATE OF ELECTROMAGNETIC TRACKING

Literature on the use of electromagnetic tracking for radiation therapy was first published in 1992 by Houdek et al.^(^ ^2^ ^)^ for stereotactic radiotherapy localization using a wired transponder sensor. This system had a sensor attached to the stereotactic halo and a source to generate the electromagnetic field, which was attached to an accessory mount. More recently in 2000, Sieler et al.^(^ ^3^ ^)^ reported on the development of a magnetic tracking technique, TULOC, for the improvement of the precision of proton radiotherapy. Their paper discussed that implantable sensors would be used to continuously monitor patient position during treatment. These sensors were miniaturized induction coils made of insulated copper wire (diameter 20 μm) wound on a piece of soft iron. The sensors had outer dimensions of 8 mm×0.8 mm diameter. Currently, information on two electromagnetic tracking systems can be found — a wired and a wireless system — both used for prostate radiotherapy.

### A. Wired system – Micropos RayPilot

**Figure 1:**
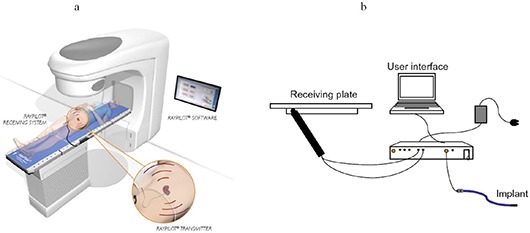
The Micropos RayPilot system: works as an add‐on (a) to existing linear accelerators and enables control of a region of interest (ROI) position throughout every radiotherapy session; consists of (b) a receiving system which is placed on the existing treatment table, the transmitter that is placed in the ROI, and the user‐interface software/computer. Figure 1(a) courtesy of Micropos Medical. Figure 1(b) reprinted from Kindblom J, et al.,(4) (Radiot Oncol.) with permission from Elsevier Inc.

The Micropos system is an active nonionizing system intended as a stand‐alone unit and needs no additional modalities such as X‐rays for determining position of the implant and target.^(^ ^4^ ^)^ With this system, a sterile dilation catheter is inserted into the penis. The internal wired antenna (implant) is located at the tip of the catheter and connected to the control unit, which converts the signal for computer evaluation (standard PC computer). The external antenna is the signal‐receiving component and consists of an integrated antennae array; it is also connected to the control unit. This external antenna is located between the patient and the couch. Kindblom et al.^(^ ^4^ ^)^ report the resolution of the tested system in the laboratory was 0.8±0.6 mm (mean ± SD). In addition to position tracking with the internal antennae, the Micropos device houses a radiation detector in order to provide an *in vivo* dose measuring using a device such as a MOSFET. Figure 1 shows a representation (Fig. 1(a)) and a schematic drawing (Fig. 1(b)) of the Micropos RayPilot system. This system has been developed only for investigational use and is not yet commercially available. Prior to its clinical use, several issues need to be addressed. First, a reliable and safe technique for transperineal implantation of the wired transponder in the prostate must be developed. Secondly, transponder stability within implanted tissue throughout the treatment course must be demonstrated. Upon completion of the radiotherapy treatment course, the transponder implant is removed and thus will not interfere with any future imaging needs, such as magnetic resonance imaging.

### B. Wireless system – calypso Medical

**Figure 2:**
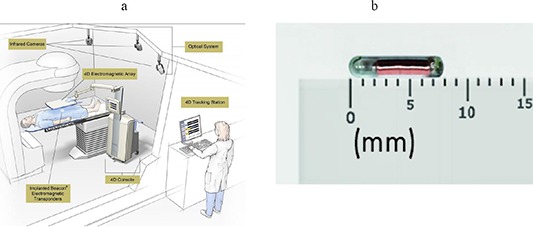
Calypso system (a) with sample beacon transponder (b). The Calypso system is positioned in the treatment room. The transponders are implanted into the patient and the array panel is positioned over the patient where it will excite and receive a signal of the beacon transponder's location in the patient. The array is positioned in the room with the use of infrared cameras mounted to the ceiling of the treatment room. Images courtesy Calypso Medical Technologies, Inc.

The Calypso Medical 4D localization system (Calypso System) utilizes radiofrequency (RF) for wireless tracking during radiation therapy. Three small (8 mm length × 2 mm diameter) beacon transponders are implanted in or near the target. Each electronic transponder consists of an AC electromagnetic resonance circuit encapsulated in glass. Localization of the transponders is achieved using an electromagnetic array consisting of four radiofrequency signaling coils and 32 receiving coils. RF signals are emitted from the array at selected pulse rates and are used to excite the transponders at their individually unique resonant frequencies. This array is located above the patient. The transponders absorb some of the radiofrequency energy and re‐emit that energy in the form of a decaying signal that is detected by the electromagnetic array. The transponder position is then detected relative to the array which, in turn, is calibrated to the room coordinate reference system by three rigidly‐mounted infrared cameras. A misalignment of the target is detected by a proprietary algorithm that identifies shifts of the target from its prescribed location anytime throughout the treatment. The accuracy of the detection of the target in phantom is less than 1 mm.^(^ ^5^ ^–^ ^11^ ^)^ Figure 2(a) shows a picture of the Calypso System and Fig. 2(b) shows an example of a single beacon transponder. The Calypso System displays real‐time graphs instantaneously highlighting shifts in position that exceed a user‐specified threshold. In order to localize and track with the Calypso System for prostate, a minimum of two transponders must be implanted, and the distance between the target centroid and the anterior surface must be <17 cm. However, centroid localization can occur at distances up to 23 cm. In cases where the prostate isocenter is beyond 23 cm, prone positioning has been found to be an acceptable alternative with the Calypso System, provided that the respiration‐induced motion is accounted for in treatment planning.

Although summarized in this manuscript, several publications have discussed, in greater detail, the clinical use, stability, and accuracy of the Calypso System.^(^ ^5^ ^–^ ^11^ ^)^ Comparisons of patient localization based on transponder locations versus stereoscopic radiographic images showed an average 3D difference of 1.5±0.9 mm.^(^ ^11^ ^)^ Submillimeter accuracy has been reported when tracking Calypso transponders moving at 3 cm/s in a volume 14cm×14cm in width and less than or equal to 27 cm away from the array.^(^ ^5^ ^,^ ^12^ ^)^ Additionally, this submillimeter localization accuracy has also been reported with studies that compared on‐board kilovoltage imaging to transponder location from isocenter.^(^ ^10^ ^)^ Significant stability of the geometry of implanted transponders throughout an entire course of external‐beam radiation therapy has been reported. The reported mean standard deviation of the intertransponder distances, calculated using transponder coordinates obtained from the CT scans and the Calypso System daily localizations, was reported to be 0.8 mm.^(^ ^7^ ^)^

Additionally, several added options are available with the Calypso System. The system has the capability of real‐time translational treatment couch adjustments that are performed as the Calypso System calculates the deviation of the transponders from the treatment isocenter. Another added option is a radiation monitoring device inside the room that makes it possible to generate position deviation reports synchronized to the radiation delivery.

## III. TECHNOLOGICAL ADVANCES FOR ELECTROMAGNETIC TRACKING

Increases in technological growth are reflected in improved methods to modulate and direct radiation beams for radiation therapy. The role of position localization and motion tracking in these methods is definitely understated. With electromagnetic tracking, explicit motion data can be recorded and harvested with a frequency of 10 Hz. The value of uninterrupted motion tracking for cancer targets is high when trying to provide precise radiotherapy to organs that move within the body independently of the surrounding tissues. This value only increases when clinicians choose to treat these organs by gating the radiation beam produced by the linear accelerator or choosing to dynamically deliver intensity‐modulated radiation therapy to these organs in order to decrease dose to normal tissues through a reduction in treatment margins. The improvements in accuracy that positional tracking can provide to radiation oncologists and physicists should help manufacturers of electromagnetic tracking systems push for advancements in their technology, as well as integration of their technologies with other major delivery systems over the next several years.

### A. Technological applications

#### A.1 Linear accelerator gating

Electromagnetic tracking in partnership with a treatment option, such as linear accelerator gating, potentially is the next major option that may arise with industrial collaborations between device manufacturers within the next ten years. Techniques currently used to gate the treatment beam from a linear accelerator utilize a variety of external surrogates to account for respiratory motion. Such devices include, but are not limited to, external marker blocks,^(^ ^13^ ^)^ infrared reflector body markers,^(^ ^14^ ^)^ as well as strain gauge belts.^(^ ^15^ ^)^ Studies have shown that the correlation between internal anatomy markers and external markers are valid, but in extremely limited cases.^(^ ^16^ ^–^ ^20^ ^)^ This correlation was reported to be poor or nonexistent unless the external surrogate measuring the skin surface was near the target volume.^(^ ^21^ ^)^ Furthermore, there is no guarantee that these correlations are constant throughout the course of radiotherapy,^(^ ^22^ ^)^ and these correlations provide the clinician little quantitative information as to the position or location of the radiation target in question. Alternative methods for tracking internal markers have focused on kilovoltage imaging. Several studies have reported on fluoroscopic imaging systems to provide accurate information of tumor motion.^(^ ^23^ ^,^ ^24^ ^)^ Real‐time tracking with systems, such as CyberKnife, allow for linear accelerator tracking of lung fiducials at a high frequency with kilovoltage imaging. However, with additional imaging there is an added expense and increased imaging dose to the patient. This is where electromagnetic tracking is most beneficial. Direct monitoring of target position may provide an avenue to gate the treatment beam without additional dose to the patient and is conducted in real time, while many imaging techniques cannot be done in real time or can be done for only a fraction of the overall treatment time. Published work has reported on the feasibility of gating based on internal position with electromagnetic tracking.^(^ ^25^ ^)^ This initial study used an electromagnetic tracking system to provide signals to a Varian Clinac via a “beam‐hold” interface of the linear accelerator in order to trigger beam on/off and gate the treatment beam. The signals for beam on/off were determined in a “gating decision box”, which compared real‐time position information with a predetermined three‐dimensional (3D) volume. ^(^ ^25^ ^)^ By gating the treatment beam this way, the clinician is able to deliver a therapeutic dose to the target volume based on absolute 3D position rather than based on phase or amplitude of a respiratory signal produced by the external surrogates for respiration.

#### A.2 Multileaf collimator tracking

**Figure 3:**
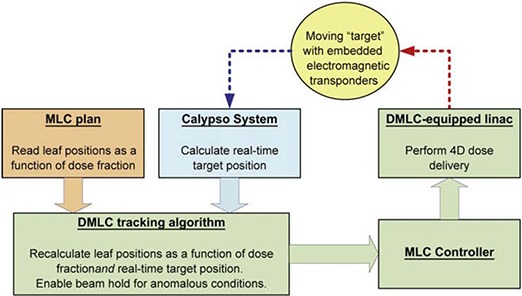
The data flow of the experimental system for multileaf collimator tracking with integrated electromagnetic tracking. Reprinted from Sawant A, et al.,^(^ ^22^ ^)^ (Int J Radiat Oncol Biol Phys.) with permission from Elsevier Inc.

As part of a multi‐institutional and industrial collaboration, a tracking system with the ability of repositioning the multileaf collimator (MLC) dynamically to follow 3D target motion in real time has been developed.^(^ ^26^ ^,^ ^27^ ^)^ The goal of MLC tracking is to dynamically find the target location and reposition the treatment beam to compensate for the target motion. This will give clinicians another technique that accounts for respiratory motion but with higher delivery efficiency than a gated treatment.^(^ ^22^ ^)^ Several investigators have published on dynamic MLC tracking of varying target motion.^(^ ^28^ ^–^ ^31^ ^)^ Electromagnetic tracking can be a key component in this effort. With electromagnetic tracking, direct measurement of the target position can be achieved (with an increased frequency of 25 Hz), while avoiding issues with fluoroscopic imaging or other imaging techniques to determine tumor position (Fig. 3).^(^ ^22^ ^)^ With the industrial collaborations between linac suppliers and electromagnetic tracking system developers, real‐time tracking with dynamic MLCs may be a viable option in the near future. Electromagnetic tracking systems provide translational position data, as well as reporting rotational information during the localization process. The rotations about the lateral, longitudinal, and vertical axes correspond to the pitch, roll, and yaw, respectively. This type of information can theoretically be implemented with dynamic MLC tracking to account for more complex motion such as in‐plane rigid rotation and target deformation.^(^ ^22^ ^)^ The combination of the MLC tracking and couch robotics integrated with an electromagnetic tracking system could potentially lead to increased conformal dose distributions and reduced dosimetric uncertainties, all with the possibility to improve treatment outcome by reducing the effects of intrafraction motion.

#### A.3 Integration with new treatment technologies

Another opportunity for electromagnetic tracking systems is integration with other radiotherapy technologies such as TomoTherapy, CyberKnife, new dynamic linear accelerators such as Varian TrueBeam, and proton facilities. Currently, electromagnetic tracking systems have been installed in standard linear accelerator rooms only, and Calypso has established master agreements with Varian and Siemens. However, integration with the treatment control systems for gating or tumor tracking, even with the mainstream linear accelerator manufacturers, has been slow in its development and clinical implementation. High precision delivery technologies could benefit from motion management systems. For example, the opportunity for CyberKnife to incorporate EM tracking with the motion of the robotic arm and linear accelerator would elevate their real‐time tumor tracking for stereotactic lung radiotherapy that currently focuses on fiducial tracking with a ceiling‐mounted kilovoltage imaging system. As another example, the effects of target motion are magnified with proton and other particle beam radiotherapy, especially if this motion is along the beam central axis. Electromagnetic tracking systems could play a large role in these facilities.

#### A.4 4D dose calculations

Several institutions have evaluated the dosimetric consequence of intrafraction prostate motion in radiation therapy, either retrospectively or prospectively. With the capability for electromagnetic tracking of prostate motion, motion can be used to calculate four‐dimensional (4D) dose distributions. At Washington University in St. Louis, investigators have developed techniques to utilize this continuous localization data provided by electromagnetic tracking to evaluate dosimetric coverage for prostate cancer patients.^(^ ^32^ ^)^ Investigators have developed a computer‐based tool to prospectively determine appropriate rotational and translational motion limits, as well as retrospectively analyze dosimetric target coverage using tracked positions of individual patient data. The investigators believe this application, referred to as SWIFTER (Semi‐Automatic Workflow using Intrafraction Fiducial‐based Tracking for Evaluation of Radiotherapy), will allow for evaluation of potentially more effective treatment techniques such as dose escalation, subprostatic boosts, and reduced‐margin treatment planning.

At MD Anderson Cancer Center Orlando, two studies have incorporated electromagnetic tracking with dose recalculation, either retrospectively or prospectively. Langen et al.^(^ ^33^ ^,^ ^34^ ^)^ studied the dosimetric impact of intrafraction prostate motion investigated for helical tomotherapy treatments. Measured electromagnetic motion tracks were used to calculate the dosimetric impact on delivered target dose distributions in prostate cancer patients. The investigators developed a retrospective dose recalculation method that allowed for the input of motion tracks from an electromagnetic tracking system and dynamically recomputed the treatment delivery for helical tomotherapy treatments. They found that for the observed patient prostate motion, the resulting dosimetric effect on respective tomotherapy plans was small.^(^ ^34^ ^)^ A second study investigated the feasibility for prospective simulation and visualization of the radiation therapy dose delivery for 3D lung tumors.^(^ ^35^ ^,^ ^36^ ^)^ These investigators propose a method of simulating and modeling lung tumors in real time and visualizing dose accumulated to them. Santhanam et al.^(^ ^36^ ^)^ further proposes that the motion tracking data will be recorded via implantable RF devices such as ones offered with electromagnetic tracking systems. They state that the possibility of real‐time dose visualization can only be realized through real‐time monitoring of patient motion with electromagnetic tracking and utilizing actual patient anatomy through 4D computed tomography.^(^ ^35^ ^,^ ^36^ ^)^

### B. Potential clinical indications

The potential clinical benefits from applications of electromagnetic tracking to clinical sites other than the prostate and prostate bed could be significant, especially in regions of large target motion, such as lung and liver. Direct measures of motion in target volumes in the thorax and abdomen will improve treatment accuracy and allow clinicians to maintain high dose gradients outside of the treatment volume to prevent excessive dose to the surrounding tissues. Several other anatomical sites also fall into this category, where organ motion may be substantial or even minimal motion may be critical. Currently, work is being done to incorporate electromagnetic tracking in several sites outside of the prostate. Hopefully, while these preliminary investigations are not yet FDA‐approved, viable options to treat these sites will become clinically available within the next several years based on this early work.

#### B.1 Lung

With the increasing use of high‐dose radiotherapy along with smaller treatment margins and the trend to use increasingly hypofractionated treatments, accurate placement of radiation fields is critical. One of the main limitations in dose escalation is the additional margin necessary to account for inter‐ and intrafraction setup errors. Respiratory motion of thoracic structures can reach up to 1.5 cm,^(^ ^37^ ^)^ and the interfraction setup error using skin marks or bony landmarks can reach up to 1 cm.^(^ ^38^ ^)^ This leads to an increase in radiation therapy volume to account for these uncertainties, and limits dose to normal tissues. Being able to directly measure the motion of tumors during breathing with electromagnetic tracking would allow clinicians to effectively reduce treatment volumes and decrease normal tissue doses without compromising tumor coverage. Observing and accounting for tumor motion from respiration during radiation treatment of non‐small cell lung carcinoma (NSCLC) has been the aim of the research proposed by several investigators, most of which are detailed in this section.

**Figure 4:**
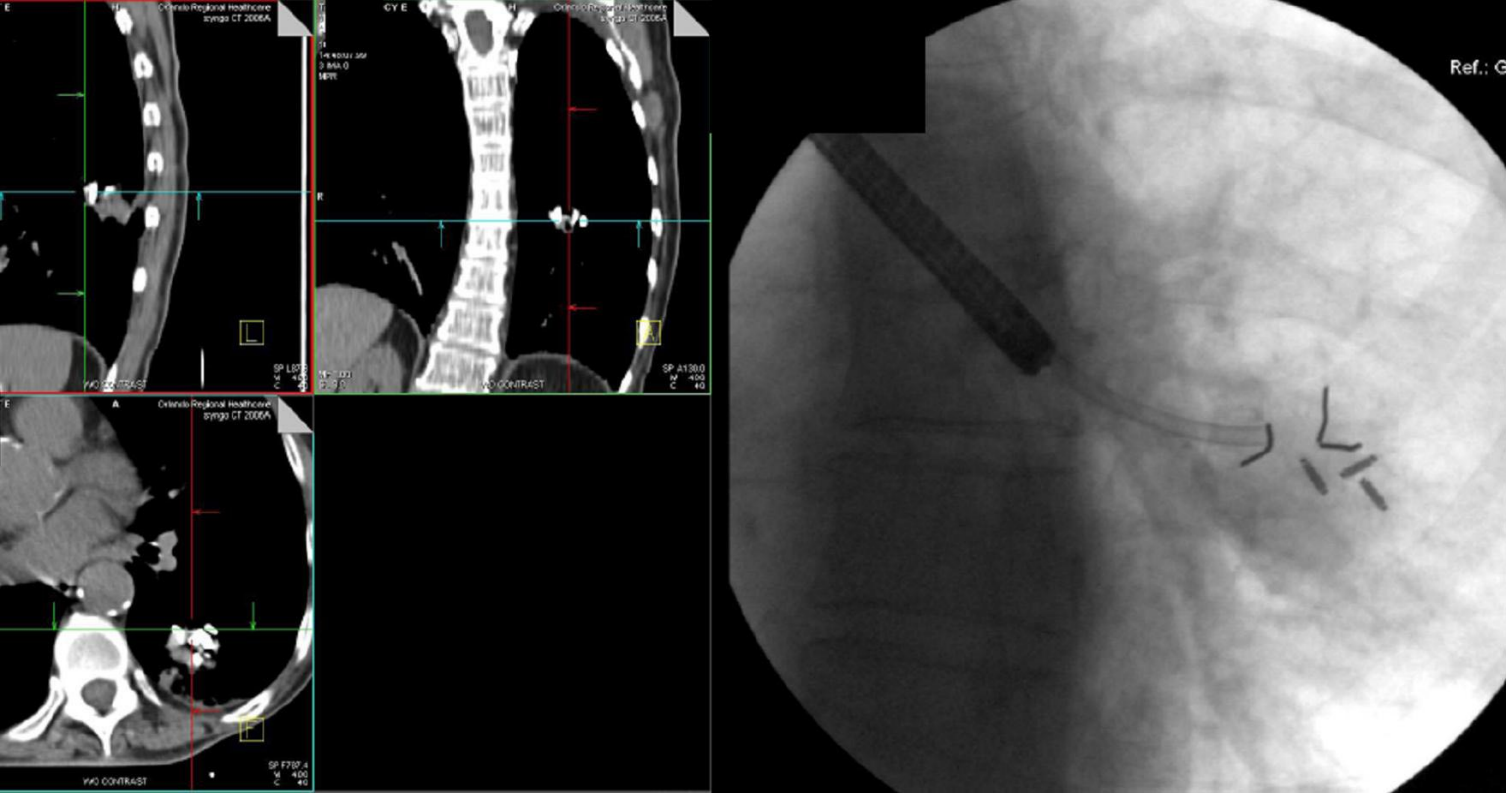
Images from lung cancer patient with left lobe, posterior lesion approximately 1.5 cm in diameter with three RF transmitters and two gold fiducial markers implanted. The fiducial markers were placed to ensure device stability. RF transmitter devices and fiducial markers imaged on (a) computed tomography and (b) a c‐arm fluoroscopic system.

**Figure 5:**
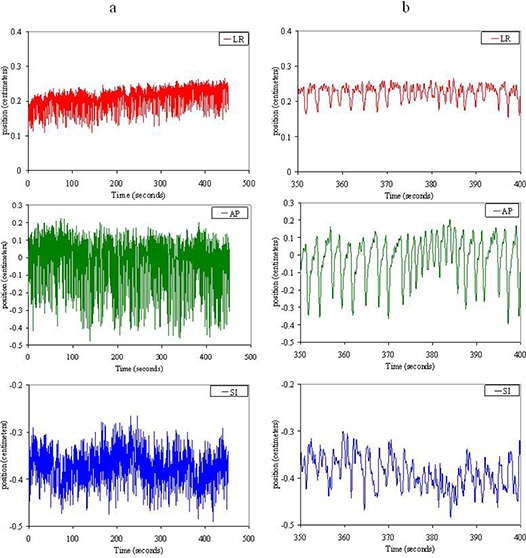
Examples of lung tumor tracks acquired through electromagnetic tracking with the Calypso system under an IRB‐approved off‐label study for NSCLC. Target motion in the same timescale is shown in the left–right (LR) (top), anterior–posterior (AP) (middle), and superior–inferior (SI) (bottom) directions for (a) the total time tracked for the single session, and (b) a detailed view of target motion data for 50 seconds. Figure 5(b) illustrates the changes in position from drift and irregular motion, which may not be realized in looking at the tracks given in Fig. 5(a), especially in the anterior–posterior direction where target motion was the largest.

At MD Anderson Cancer Center Orlando, preliminary work has been done to directly measure tumor motion using an electromagnetic tracking system under an Institutional Review Board (IRB)‐approved off‐label study for NSCLC.^(^ ^39^ ^)^ Additionally, this study requires investigational device exemption (IDE) from the FDA. To date, a total of twelve RF transmitters have been implanted into five patients with varied success. Bronchoscopic implantation of the RF devices was performed along with implantation of gold fiducial markers to improve stability and fixation (for every RF transmitter device, one fiducial marker). The lung implantation was performed using the superDimension system (superDimension Inc., Minneapolis, MN) that is designed to allow CT‐guided bronchial navigation for biopsy, but is used at our clinic for implanting markers^(^ ^1^ ^)^ as well as RF transmitters in and around lung lesions (Fig. 4). Of the twelve implanted RF devices, three did not remain fixated in the tumor location. Motion data were collected for all enrolled patients. The authors reported that this study was meant to collect real‐time tumor motion tracks before and after treatment and not to interfere with the standard of care for radiotherapy treatment for the patients enrolled in the investigation at their clinic. Figure 5 displays an example of the target tracks obtained for one patient during one session.

**Figure 6:**
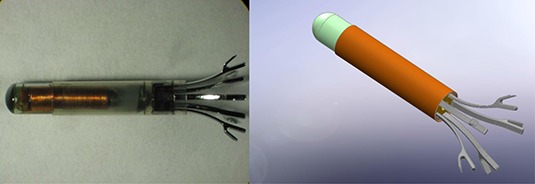
Anchored lung transponder from Calypso Medical Technologies Inc. The lung transponder is intended for bronchoscopic implantation in small diameter airways. The anchored transponder consists of the prostate transponder with a 5‐legged nitinol stability feature. Images courtesy Calypso Medical Technologies.

In a move toward clinical availability for electromagnetic tracking within the lung, an electromagnetic tracking device with a stabilization feature has been developed by Calypso Medical and investigated by a group at Washington University using a canine model.^(^ ^40^ ^,^ ^41^ ^)^ Mayse et al.^(^ ^42^ ^)^ reported successful bronchoscopic implantation for all 54 of the stabilized beacon transponders. At 60 days follow‐up, all 15 of the transponders with working electromagnetic cores had stable intertransponder distances as measured by the Calypso System. The stabilization feature incorporating five nitinol legs was created to house the electromagnetic beacon transponder (Fig. 6). The nitinol legs are designed to deploy into the bronchus after bronchoscopic implantation. The authors reported that successful implantation and fixation were very sensitive to the implantation technique, in that too much force on the implantation catheter may lead to pneumothorax and not enough force may lead to a low rate of fixation.

#### B.2 Liver/pancreas

Implanted fiducials are commonly used for image‐guided radiation therapy (IGRT) of abdominal tumors, especially in stereotactic body radiation therapy (SBRT) cases.^(^ ^43^ ^,^ ^44^ ^)^ Fiducial implantation is often performed as an out‐patient procedure, with little migration^(^ ^45^ ^)^ and clear compatibility with kilovoltage imaging; thus, its value for treating moving targets such as liver tumors far outweighs its cost. Furthermore, the introduction of electromagnetic tracking for these targets could potentially be quite valuable. There are few studies that have investigated the use of electromagnetic tracking in abdominal targets with the radiofrequency devices. This may be due to a limitation in implantation techniques for the relatively large diameter devices that are currently available on the market, as well as the corresponding concerns with RF device migration after implantation. Another concern with implantation of the RF transmitter devices with targets such as the liver is the need for post‐treatment magnetic resonance imaging (MRI) studies for patient follow‐up. The potential for increased image artifact created by the implanted RF transmitters within an MRI study would prove too costly for clinicians. This issue is further explained below with regard to RF transmitter size and the need for removable RF devices. At the University of Pennsylvania, investigators have implanted RF transmitters into the pancreas of three patients under laparoscopic guidance.^(^ ^46^ ^,^ ^47^ ^)^ The investigators have reported stability with the RF device implantation and all patients were without complications.

#### B.3 Breast

Reduction of margins, setup error, and the possibility of intrafraction motion during partial breast irradiation are all factors that may be improved with the introduction of an electromagnetic tracking system. Preliminary work has been done to test the feasibility of electromagnetic localization for external beam partial breast irradiation.^(^ ^48^ ^)^ Investigators at Swedish Medical Center in Seattle have used an electromagnetic tracking system under an IRB‐approved protocol to implant RF devices into the lumpectomy cavity of fifteen patients.^(^ ^49^ ^)^ Implantation of electromagnetic transmitters may not be ideal for breast cancer patients due to the need for post‐treatment magnetic resonance imaging studies for clinical follow‐up. Therefore, the investigators took precautions to implant the RF devices temporarily via interstitial catheters and removed them following course of radiotherapy treatment.

#### B.4 Cervix

Investigators at Emory University have recently started a clinical trial to evaluate intrafractional cervical motion utilizing an electromagnetic tracking system in cervical cancer patients. The trial states that two RF devices will be temporarily placed within the cervical os via sutures and tracked during the course of radiation therapy treatment. The patients will continue with the clinic's standard of care, but the RF transmitter positions will be continuously tracked and recorded. The end goals for the investigators are to improve the current knowledge of cervical motion and tumor regression during radiation therapy with the use of electromagnetic tracking.^(^ ^50^ ^)^

#### B.5 Central nervous system

**Figure 7:**
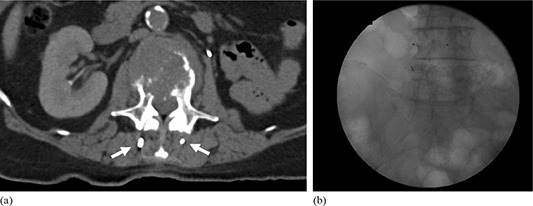
Images from patient who presented with painful bony metastases to the fourth lumbar vertebral body with three RF transmitter devices surgically implanted into the paraspinal muscle for single‐fraction stereotactic body radiotherapy for pain relief and quality of life enhancement. RF transmitters imaged on (a) computed tomography and (b) a c‐arm fluoroscopic system.

**Figure 8:**
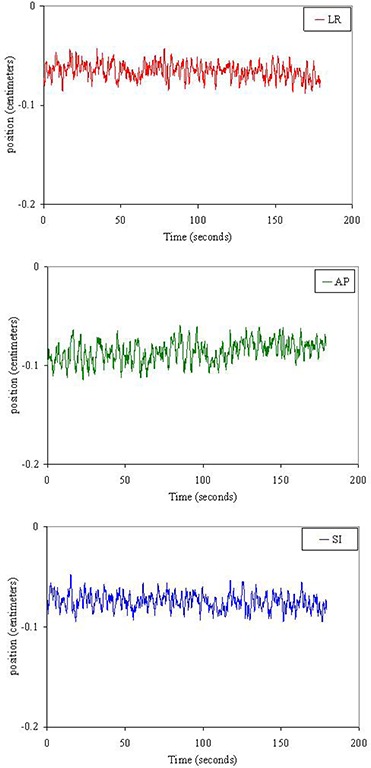
Examples of target tracks acquired through electromagnetic tracking with the Calypso system under an IRB‐approved off‐label study for spine stereotactic body radiotherapy. Target motion in the same timescale is shown in the left–right (LR) (top), anterior–posterior (AP) (middle), and superior–inferior (SI) (bottom) directions for the total time tracked before the single fraction treatment. In this example, the target motion in each direction was less than 2 mm.

Although SBRT is not restricted to the radiotherapy for the central nervous system, it has become more common in radiation oncology, and IGRT has become a necessary component of this type of treatment. Electromagnetic tracking could easily play a critical role in the delivery of SBRT for CNS‐related systems. The continuous monitoring of target displacements could provide an improved means to deliver accurate dose to the target while reducing dose to normal tissues, especially in cases where the target could move during the treatment delivery. In one such example, Willoughby et al.^(^ ^39^ ^)^ reported on the use of electromagnetic tracking during spinal radiosurgery. These investigators, under fluoroscopic guidance, transcutaneously implanted RF transmitters into the paraspinal muscles at the level of the targeted vertebra in six patients (Fig. 7). The implantation was successful in all six of the spine patients. There were no complications related to the implantation processes. All patients had at least two CT scans prior to treatment delivery to confirm stability and geometry of the RF devices. At the time of treatment, all patients also had kV X‐rays to confirm stability and geometry in the treatment position prior to each delivery. Similar to the liver, issues with post‐treatment magnetic resonance imaging studies for the spine need to be mentioned and will be addressed in more detail below. Tracking data on the available electromagnetic tracking system was obtained on all four patients. An example of this tracking data from one patient can be seen in Fig. 8. A second scenario could also be envisioned for stereotactic radiosurgery patients. Currently, many institutions have gone to frameless radiosurgery using fiducials and optical tracking techniques. Accordingly, an interesting question arises if this could be done using electromagnetic tracking, as proposed in 1992 by Houdek et al.,^(^ ^2^ ^)^ for intracranial lesions. For example, RF transmitters could be implanted into a bite‐block or similar device, and cranial displacements could be tracked and recorded with tolerances set to gate the radiation beam off as increases to translation or rotational motion occurs.^(^ ^51^ ^)^

### IV. CHALLENGES TO ELECTROMAGNETIC TRACKING

Electromagnetic tracking is still an immature technology in radiation oncology, and as such, the clinical applications and clinical experience are both very limited. Some of the major challenges with incorporating electromagnetic tracking into daily use for radiation therapy are due to the limited number of available technologies. With the Calypso System being the sole FDA‐approved electromagnetic tracking system used in radiation oncology, development resources are limited. Hence, there are many opportunities for improvement over the next decade.

First and foremost among limitations, electromagnetic tracking has only been approved for use in prostate cancer treatment. Any use at other disease sites must be approved and monitored by the FDA and the local IRB. While there is utility in tracking the prostate, there is more potential benefit at other disease sites mentioned previously. The development of techniques to treat these clinical sites, along with the regulatory approval to do so, needs to be addressed in the near term.

Some technical challenges that are inherent to electromagnetic tracking systems are related to the electromagnetic array. In the currently available electromagnetic tracking system, the array rests directly above the prostate cancer patient with a need for direct line of site to the infrared cameras that track the array's motion and position relative to isocenter. The combination of the array's position and RF transponder's position relative to the array, provides the electromagnetic tracking system with the actual room coordinate system and, further, the transponder's position relative to isocenter. However, several issues arise due to the presence of the electromagnetic array, such as issues with couch angles, distance issues from the array to the implanted RF devices, and collision issues with the gantry due to the proximity of the array to the gantry head. If the need for the array (anterior to the patient) was not there or if the array could be in another location (such as embedded in the treatment couch), many of these issues could be resolved and the infrared cameras in the room could be removed. Although for this to happen, treatment couches would be required to maintain millimeter‐scale positioning accuracy, or maintain the infrared tracking system but tied directly to the treatment couch. Another issue with the electromagnetic tracking systems is in the limited field of view (FOV) with the array. The current FOV limitation of the available system is not a major problem when treating the prostate (the system's intended purpose); however, if new clinical indications are to be implemented, this limitation will need to be addressed. Even more important, if the flat‐field generator were placed in a location adjacent to the patient with no interference from electrical and ferrous objects in the accelerator room, the benefit of tracking implanted needles or devices would be infinite.

Another challenge to electromagnetic tracking systems is regarding the RF device size. With the currently available RF transmitter device, its large size allows for high signal to noise. However, the 8 mm length× 2 mm diameter device size requires a 14‐gauge needle for transperineal implantation, which is considered large for transcutaneous needle insertion for areas such as the liver or bronchoscopic insertion into the lung. The risk of pneumothorax with transcutaneous approaches is estimated to be in the 20%–30% range, and even higher (40%–50%) in patients with obstructive airway disease.^(^ ^1^ ^)^ The risk of pneumothorax is greatly reduced with transbronchial fiducial placement in the lung, even in peripheral lesions under fluoroscopic guidance.^(^ ^1^ ^)^ Also, when taking into consideration follow‐up imaging studies of patients, the RF transmitter's relatively large ferrite core creates significant artifact on MRI or even kilovoltage cone beam CT.^(^ ^52^ ^)^ It was reported that implanted transponder displacements due to the MR field were minimal; however, the null signal reported due to image artifacts were up to 1.5 cm in radius and 4 cm in length, thus creating significant issues with post‐treatment imaging with patient follow‐up.^(^ ^52^ ^)^ A reduction in the sizes of RF devices would be beneficial, but would not prevent these image artifacts from occurring. Development of removable RF transmitters would be the next expected course of action for treatment areas that benefit from post‐treatment MR imaging.

Another challenge is to provide a system only for motion tracking, not localization and tracking. In the current configuration of electromagnetic tracking systems, there is great care to ensure that the RF transmitter devices are in the correct location relative to the isocenter. In order to perform this calculation accurately and precisely there are limitations to the additional noise from metal or other devices in the room, restrictions on the implantation geometry, as well as procedures to limit the amount of motion the RF devices are experiencing in the implantation location. If tracking‐only were allowed, the user would be responsible to localize the patient via some other imaging modality, and the electromagnetic tracking system would gain flexibility by providing only relative coordinates of the implanted RF transmitters. This could be used in computed tomography for 4D CT, or used during treatment in other sites in the body where localization issues based on single implanted markers may arise due to intrafraction motion and deformation, such as lung and liver. Also, if relative displacement numbers were reported, the need for multiple implanted RF devices may not be necessary. Multiple transmitters, if implanted correctly, provide the clinician with rotational information and possible organ deformation information; however, without imaging, it cannot be known if the reported angles are due to marker migration or due to organ deformation. If rotational information is not desired, then multiple RF devices may not be needed.

Another challenge to electromagnetic tracking is with competing technologies that may provide tracking via a different delivery system. Although radiofrequency‐based electromagnetic tracking systems can be used in real time to track tumor location due to its fast update rate, new emerging technologies are constantly being developed that may provide a similar tracking mechanism. One such technology being developed by Navotek Inc. (Navotek Inc., Israel) provides the ability to track targets within the body. Navotek is developing a gantry‐mounted radioactive fiducial tracking system that is reported to provide submillimeter accuracy for patient localization and monitoring.^(^ ^53^ ^)^ Radioactive fiducial tracking, though not FDA‐approved, challenges technologies such as electromagnetic tracking with technical advancements in implantable radioactive materials and localization of these sources. However, this technology may have insufficiencies of its own such as the lack of ability to provide rotational information and possible organ deformation information. In addition, if any migration of the radioactive source does occur, it will be quite difficult to determine by how much and to where it is migrating.

The last challenge to electromagnetic tracking is with reimbursement. While not a technological limitation, the up‐front capital expense of electromagnetic tracking systems and the ongoing expense of the implantable markers remain barriers to widespread implementation of these technologies. Because of the high cost of these RF transmitters and the varying levels of reimbursement in different geographical regions, it is cost prohibitive for many centers to consider this technology for routine clinical use. Unless these cost and reimbursement issues are addressed, electromagnetic tracking technology may not experience further growth in radiation oncology. Continued research collaborations need to show that (a) electromagnetic tracking is a growing technology with increased development of treatment options, (b) proper and unbiased efficacy tests show an improved therapeutic effect, and (c) effectiveness tests show electromagnetic tracking does help in localization and targeting of the tumor region. For example, the latter two points could be tackled through dosimetric studies that show intrafraction motion during radiotherapy does result in differences in the delivered dose versus the planned dose — similar to some of the studies mentioned above related to retrospective dose recalculations.^(^ ^33^ ^,^ ^34^ ^)^

### V. SUMMARY

Electromagnetic tracking systems enable accurate patient set‐up, respiratory correlated radiotherapy, collision avoidance, and adaptive radiation therapy. Furthermore, daily use of these systems is minimally invasive and delivers no additional ionizing radiation to the patient. Due to these advantages, electromagnetic tracking systems are expected to play a continued role in improving the precision of radiation delivery. There are a number of technical and fiscal issues that need to be addressed in the near term, however, to ensure the success of these technologies in improving patient care over the next ten years and beyond.

## ACKNOWLEDGMENTS

The authors would like to thank Calypso Medical Technologies and Micropos Medical for their assistance in writing this manuscript.
